# ML assisted optimization of DR based MIMO antenna for 5G communication systems

**DOI:** 10.1038/s41598-026-45334-2

**Published:** 2026-04-10

**Authors:** Sumit, Vinay Kumar, Ashish Pandey, Anand Sharma, Pinku Ranjan, Rajeev Tripathi

**Affiliations:** 1 Department of Electronics and Communication Engineering, MNNIT Allahabad, Prayagraj, Uttar Pradesh India; 2 Department of Data Science and Engineering, Manipal University Jaipur, Jaipur, Rajasthan India; 3 Department of Electrical and Electronics Engineering, ABV-Indian Institute of Information Technology and Management (IIITM), Gwalior, Madhya Pradesh India

**Keywords:** Millimetre-wave, Dielectric resonator antenna, MIMO, Metasurface, Mutual coupling reduction, Machine learning, Surrogate modelling, Engineering, Mathematics and computing

## Abstract

This work presents a dual-port cylindrical dielectric resonator antenna (CDRA) based MIMO design for 27-GHz 5G communication, assisted by a suspended complementary split-ring resonator (CSRR) metasurface for port decoupling. The antenna is realised on Rogers RT Duroid 5880 substrate of thickness 0.254 mm, and the cylindrical dielectric resonator uses Rogers RT Duroid 6010 material. In simulation, the metasurface improves the mutual coupling from about − 27.56 dB to − 45.22 dB at 27 GHz (≈ 17.6 dB enhancement) while maintaining impedance matching. A prototype is fabricated, and measurements confirm an operating band of 26.24–27.94 GHz and show clear isolation improvement when the metasurface is applied, with a peak realised gain of around 5 dBi in the band. To reduce optimization time at millimetre-wave frequencies, a dataset generated in HFSS (19,100 samples) is used to train surrogate regression models, including Decision Tree, K-Nearest Neighbours, Random Forest, Extreme Gradient Boosting, and a Deep Neural Network (DNN), to predict S_11_ and S_12_ from geometry parameters and frequency. Among the studied models, the DNN gives the best prediction for S_11_ (R^2^ = 0.991), while Random Forest and DNN show the best performance for S_12_ (R^2^ = 0.993 and 0.990). The combined antenna design and surrogate modelling approach supports faster parameter search for high-isolation mmWave DRA-MIMO antennas.

## Introduction

Fifth generation (5G) communication systems are expected to support high data-rate links and low-latency services, which has encouraged the use of millimetre-wave (mmWave) spectrum. At these frequencies, multiple-input multiple-output (MIMO) antennas are widely studied to improve link capacity and reliability. However, compact mmWave MIMO implementations often suffer from strong mutual coupling between antenna elements, which degrades isolation and limits diversity performance^1^.

Dielectric resonator antennas (DRAs) are suitable candidates for mmWave operation because the radiator does not rely on a large metallic patch, which helps in reducing conductor loss at high frequencies^2^. Several techniques have been reported to enhance isolation in DRA-based MIMO antennas. For example, a frequency selective surface wall was placed between DR elements to reduce coupling^3^, metallic vias were introduced to suppress interaction between ports^4^, and metallic strips were used to improve isolation in dual-port DRA configurations^5^. Metasurface-inspired structures have also been explored for mmWave decoupling^6^, and arrangement-based approaches such as antiparallel excitation have been used to improve isolation^7^. While these methods demonstrate improvements, compact dual-port mmWave designs still require stronger isolation with a simple and fabrication-friendly structure, particularly when the spacing between antenna elements is limited.

In parallel, antenna optimization at mmWave frequencies becomes time-consuming because full-wave electromagnetic simulations require fine meshing and dense frequency sampling. Machine learning (ML) based surrogate modelling has therefore emerged as a practical approach to reduce optimization time by learning the relationship between design parameters and antenna responses. Prior studies have used ML models to optimize microstrip and printed antennas^8^, ultra-wideband monopole antennas^9^, and dielectric resonator antennas in selected settings^10,11^. However, ML-assisted optimization for mmWave dual-port DRA-MIMO antennas combined with a suspended metasurface decoupling structure has not been sufficiently established in open literature, particularly with a clear workflow that links (i) electromagnetic design, (ii) dataset construction, (iii) surrogate model training, and (iv) verification through measurements.

This paper addresses this requirement by presenting a dual-port cylindrical DRA-MIMO antenna operating around 27 GHz, where a suspended complementary split-ring resonator (CSRR) metasurface is used to suppress mutual coupling without disturbing impedance matching. A data-driven surrogate modelling framework is then applied to predict |S_11_| and |S_12_| from key geometry parameters and frequency, enabling faster parameter search compared to repeated full-wave optimization. The proposed approach is validated through fabrication and measurement, and the diversity characteristics are also reported.

### Paper organization

Section "Related work and research gap" summarizes related work and highlights the identified gap. Section "Antenna design layout and unit cell" describes the antenna configuration and the metasurface unit cell. Section "Proposed antenna analysis" discusses the antenna operating mechanism and the improvement of isolation with the suspended metasurface. Section "Machine learning optimization" presents dataset generation and ML-based surrogate modelling with performance comparison. Section "Measurement setup for proposed antenna" describes the measurement setup. Section "Experimental outcome and diversity parameter" reports the measured results and diversity parameters. Section "Conclusion" concludes the paper.

## Related work and research gap

High isolation mmWave MIMO antennas have been reported using several decoupling approaches such as frequency selective structures, vias, metallic strips, and engineered surfaces. In DRA-based mmWave MIMO systems, isolation improvement has been demonstrated using an FSS wall^3^, metallic vias inside the resonator^4^, and metallic strip loading^5^. Metasurface- and metamaterial-inspired structures have also been explored for isolation enhancement at mmWave^6^, while arrangement-based excitation, for example antiparallel feeding, has been used to improve decoupling in dual-port DRA configurations^7^. Although these studies show measurable isolation improvement, compact dual-port mmWave DRA designs still require decoupling structures that are simple to fabricate and sufficiently stable under practical assembly constraints.

Recent studies continue to report advances in mmWave DRA-MIMO and decoupling, including compact designs that use additional metallic features and careful radiator/feed tuning to improve bandwidth and isolation^12^. In parallel, engineered surfaces, including metasurface and superstrate concepts, remain an active direction for improving MIMO behaviour by controlling near-field interaction and surface currents, with recent mmWave examples showing that metasurfaces can improve MIMO performance when spacing and alignment are properly controlled^13,14^. However, many of these solutions still involve multiple interacting variables, and their measured performance can be sensitive to alignment, air-gap accuracy, bonding/fixtures, and fabrication tolerances, which is especially important around 26–28 GHz.

Alongside electromagnetic decoupling, optimization at mmWave frequencies is often limited by the high computational cost of full-wave simulations. For this reason, ML-driven surrogate modelling has become a practical tool to learn antenna responses from sampled simulations and guide parameter search at lower cost. Earlier studies applied ML algorithms to optimize printed antennas^8,9^, and initial attempts have also been reported for DRA optimization using ML or hybrid optimization methods^10,11^. More recent works emphasize surrogate-assisted optimization and multi-parameter optimization frameworks for antennas, showing that surrogate learning and evolutionary search can reduce design cycles and handle costly simulations more efficiently^15–18^.

Recent papers also indicate that metasurface-based decoupling and data-driven optimization are beginning to converge in MIMO antenna research. Narayanaswamy et al. reported a machine-learning-aided tapered four-port MIMO antenna for V2X communication, where learning models were used to support parameter tuning for gain and isolation improvement^19^. In another work, Narayanaswamy et al. proposed a wideband dual-port MIMO antenna with a metasurface absorber, where deep learning was used to optimize the absorber dimensions and improve port isolation in the millimetre-wave band^20^. Saha et al. presented an R-shaped microstrip patch antenna for mm-wave 5G and used CSO with curve fitting to reduce manual tuning effort during optimization^21^. Kumar et al. reported a compact four-port multiband circularly polarised mm-wave MIMO antenna for wearable and beyond-5G applications, highlighting the present trend towards compact multiport systems^22^. Ali et al. designed a 25 to 30 GHz antenna array using interlinked CSRR loading and defected ground features and reported improvement in impedance behaviour and isolation with practical hardware validation^23^. Elabd et al. proposed a wideband four-port mm-wave MIMO antenna in which a metasurface and an FSS layer were used together to suppress mutual coupling and enhance radiation performance across 24 to 50 GHz^24^. In addition, recent decoupling work on two-port DRA-MIMO using defected ground structures (DGS) highlights that multiple practical decoupling options remain relevant, but they must be assessed with measured validation and clear reporting of tolerances^25^.

These studies collectively support the relevance of both metasurface-based decoupling and data-driven optimization. However, clear opportunities remain for compact mmWave DRA-MIMO systems, particularly when practical fabrication constraints and computational cost must both be addressed.

*Research gap:* Two gaps are directly relevant to compact mmWave DRA-MIMO systems.

1. For suspended metasurface decoupling in dual-port mmWave DRA configurations, there is still a need for designs that clearly report (a) baseline coupling, (b) final coupling, (c) isolation improvement, and (d) measured-versus-simulated differences with practical reasons.
2. For ML-assisted optimization, many studies focus mainly on prediction accuracy, but fewer present a complete and reproducible workflow for mmWave DRA-MIMO decoupling that includes clear optimization objectives, model reasoning, and verification against HFSS and measurements.

**Fig. 1:**
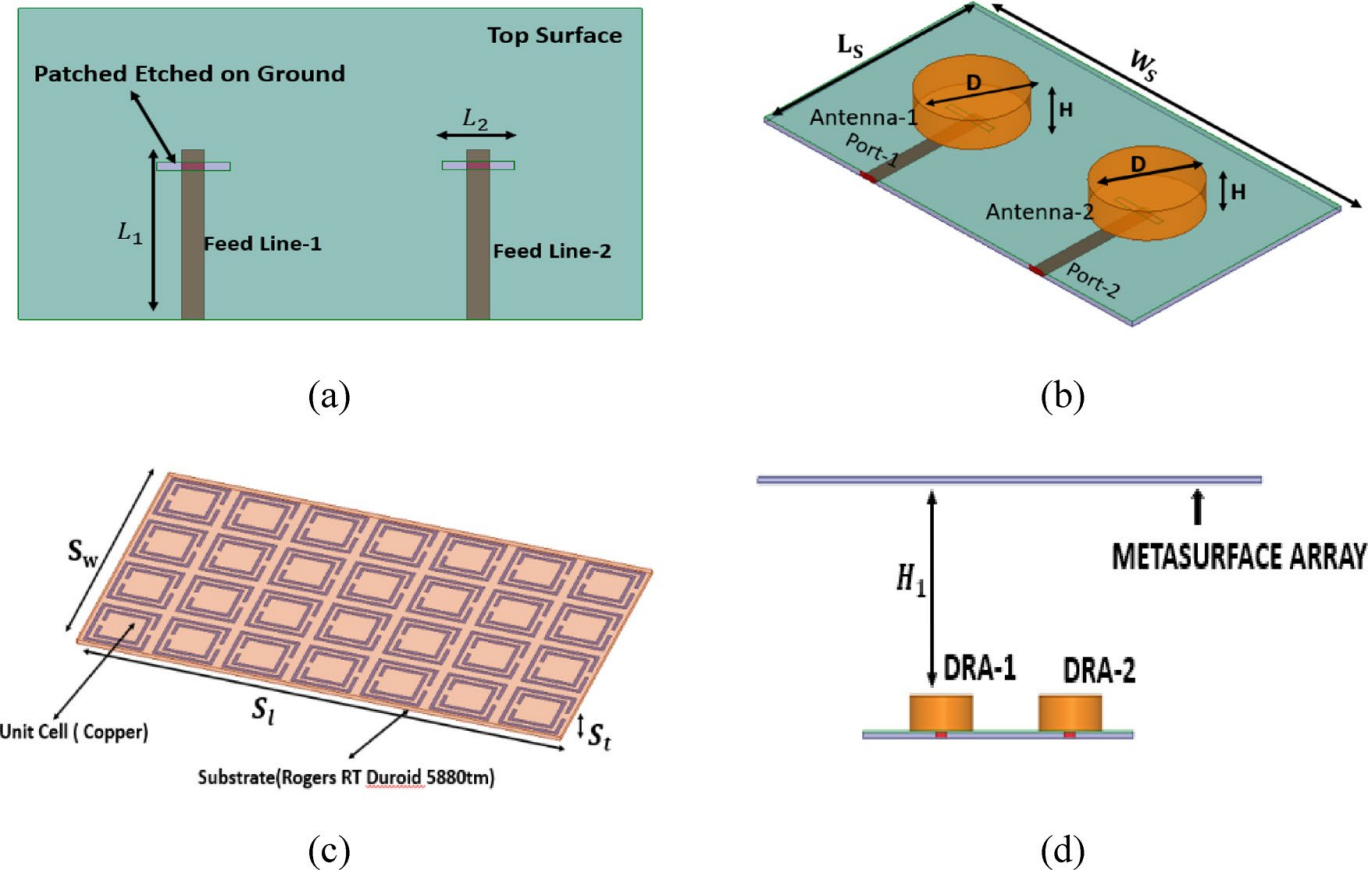
Structural view of the proposed MIMO Antenna; (**a**) Top view. (**b**) Panoramic View (with DR placed). (**c**) 4 × 7 metasurface array. (**d**) Side view of antenna with metasurface on top.

**Fig. 2:**
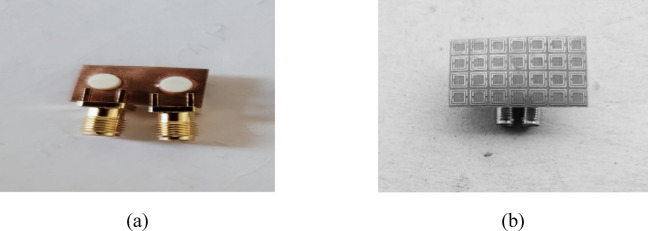
Fabricated MIMO antenna (**a**) Without Metasurface. (**b**) With Metasurface.

**Fig. 3:**
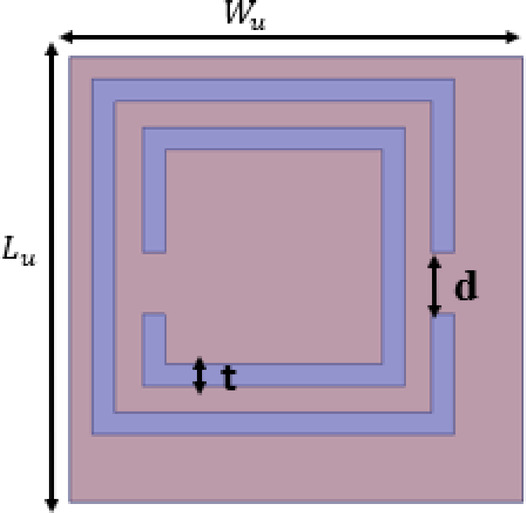
Design of unit cell.

**Fig. 4:**
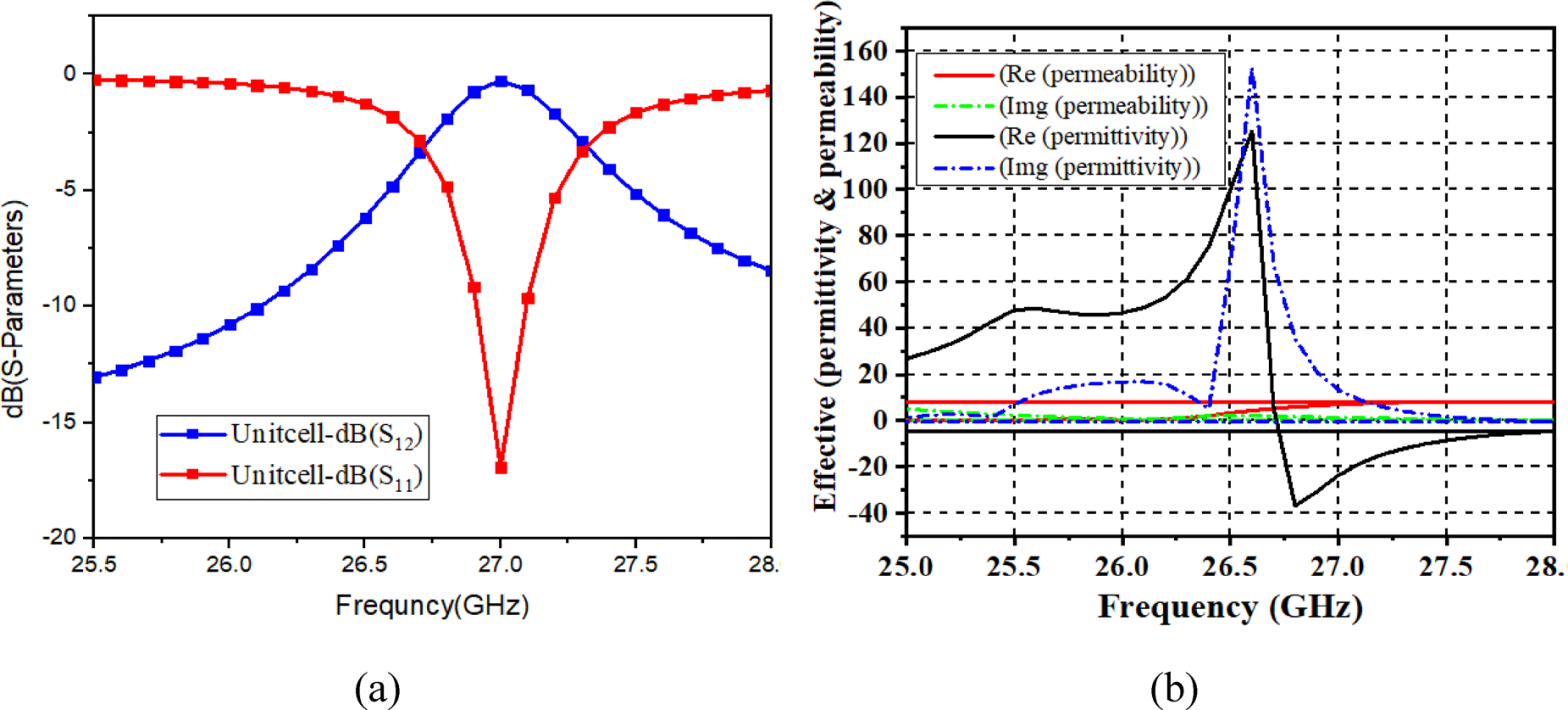
(**a**) S-parameter curves of unit cell. (**b**) Extracted parameters of the Unit cell.

**Fig. 5:**
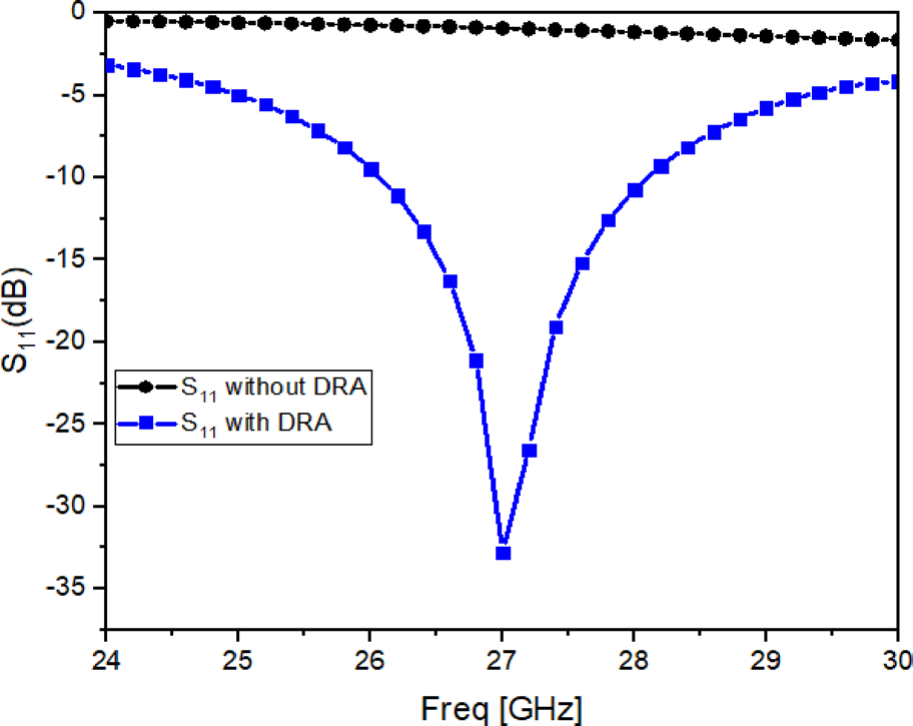
Reflection coefficient (S_11_) with and without single antenna.

**Fig. 6:**
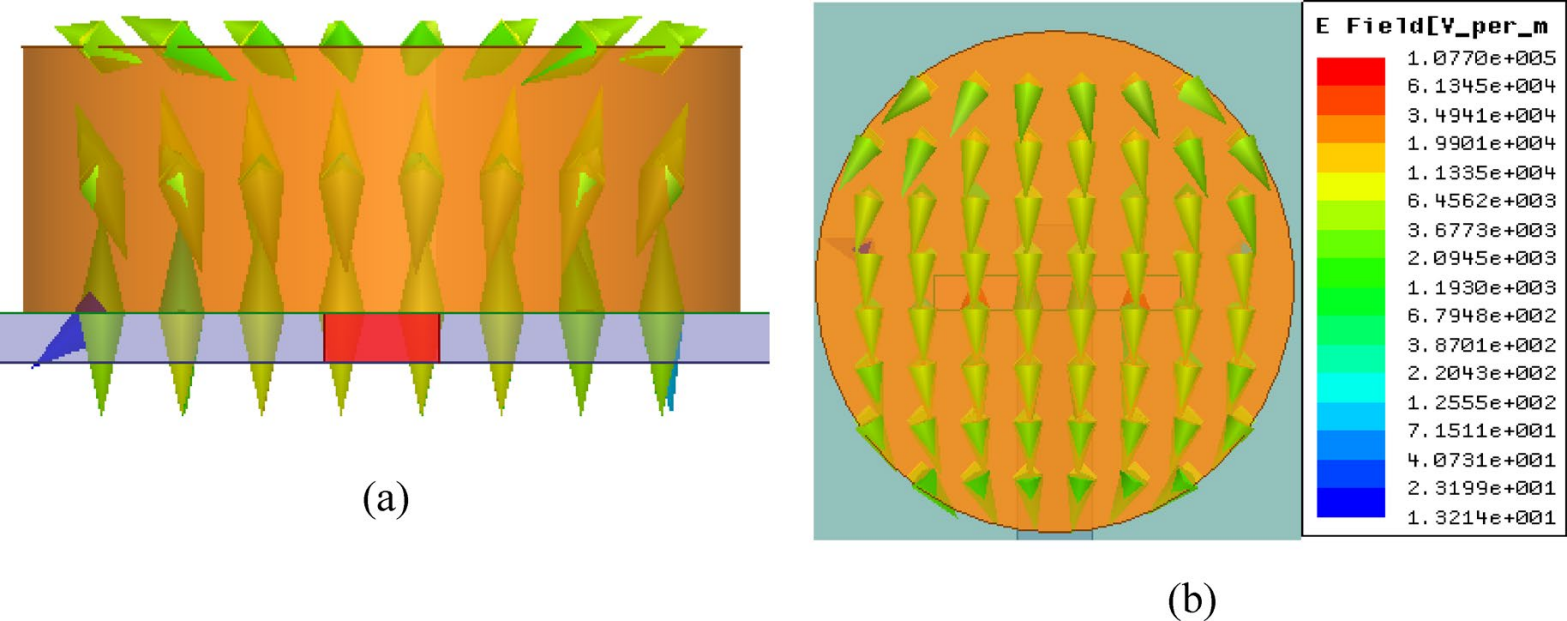
E-field variation on Single DRA at 27 GHz. (**a**) Top view. (**b**) Side View.

**Fig. 7:**
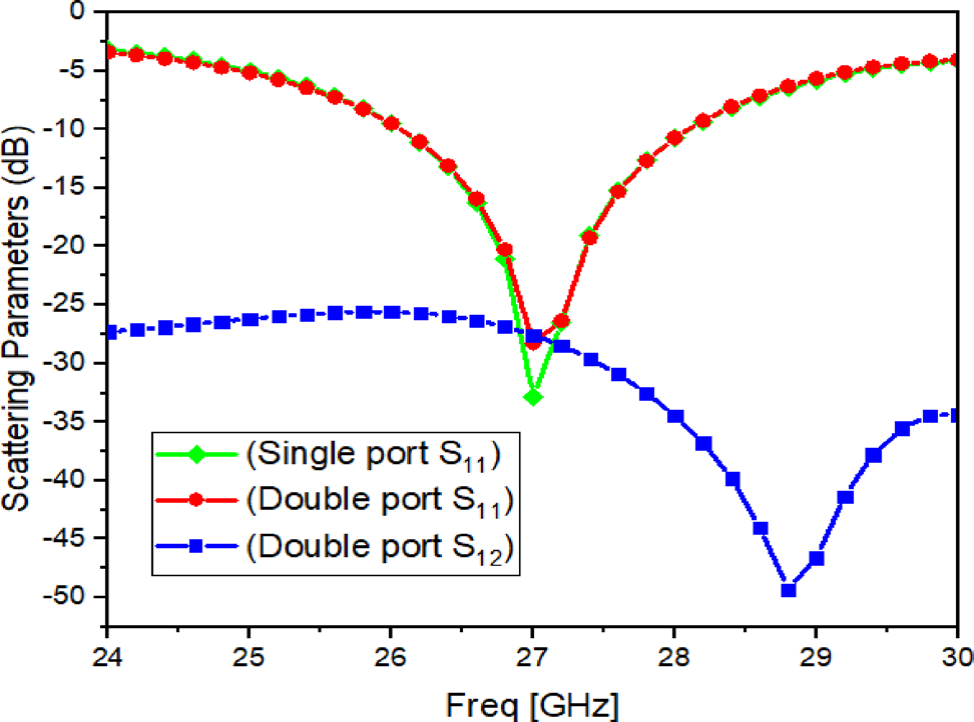
Reflection coefficient (S_11_) and Transmission coefficient (S_12_) of MIMO Antenna with single and double port.

**Fig. 8:**
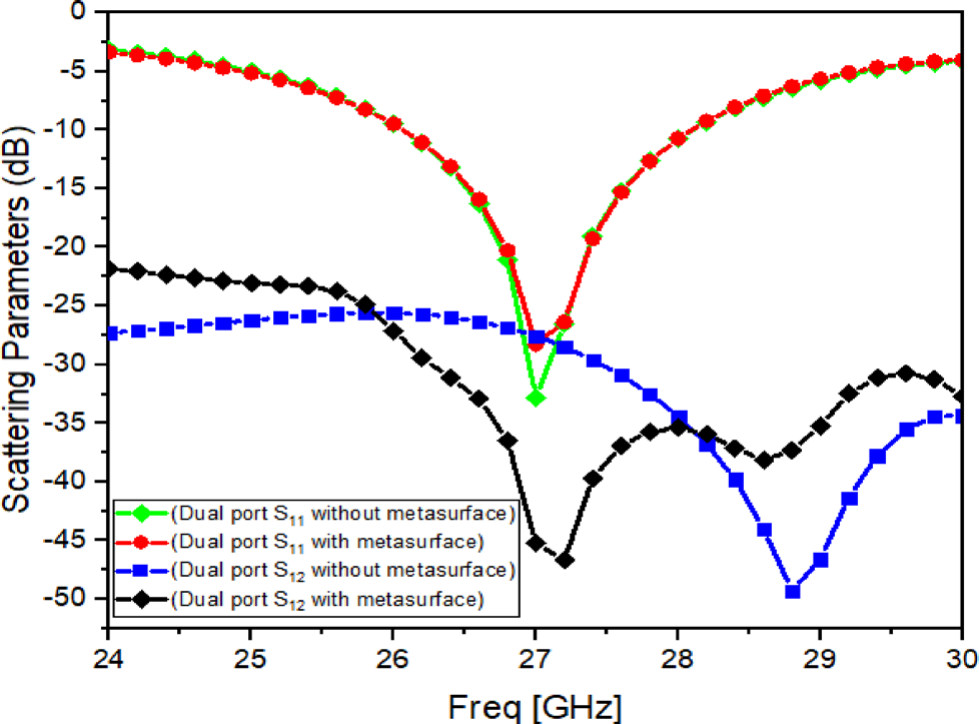
Reflection coefficient (S_11_) and Transmission coefficient (S_12_) with and without Metasurface.

**Fig. 9:**
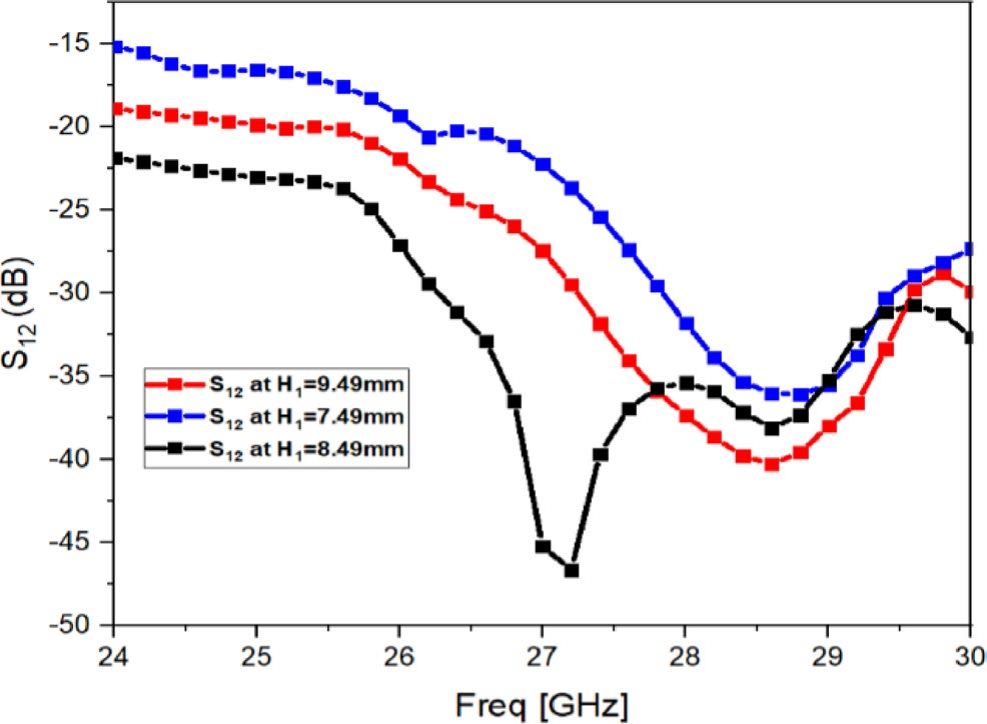
Transmission coefficient (S_12_) with distance varying between Antenna and Metasurface.

**Fig. 10:**
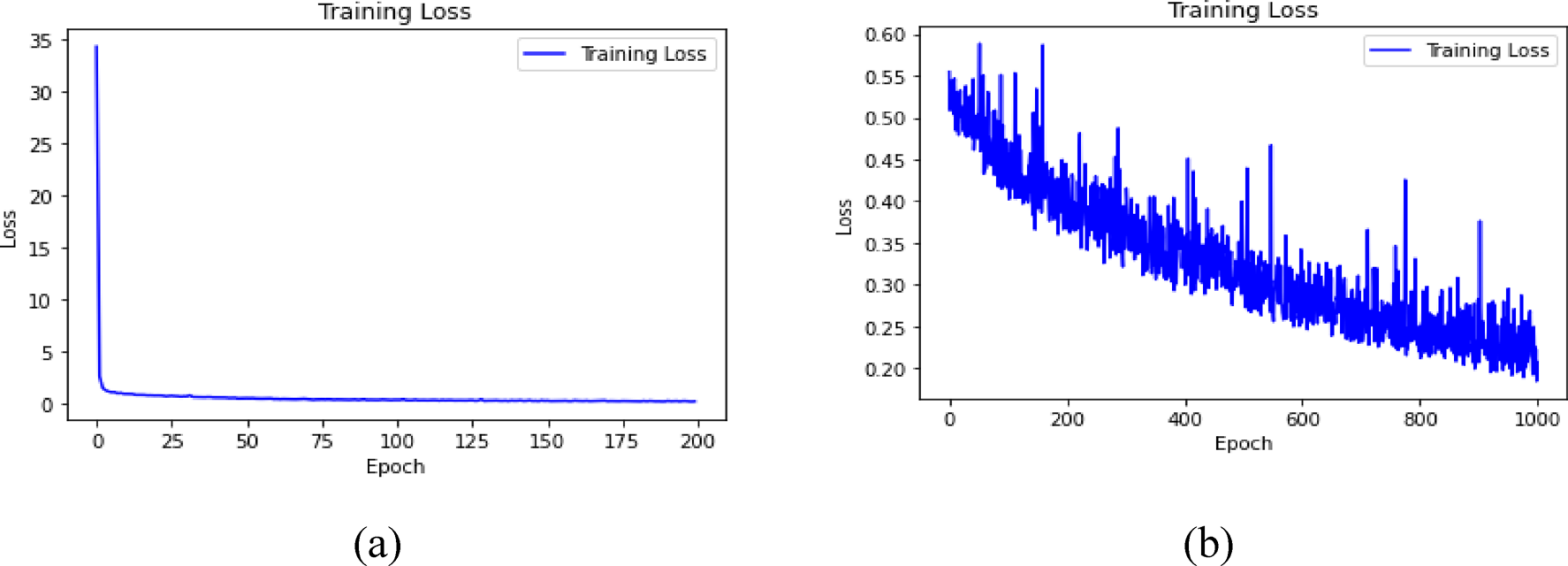
Epoch vs. Training Loss for DNN (**a**) for S_11_ (**b**) for S_12_.

**Fig. 11:**
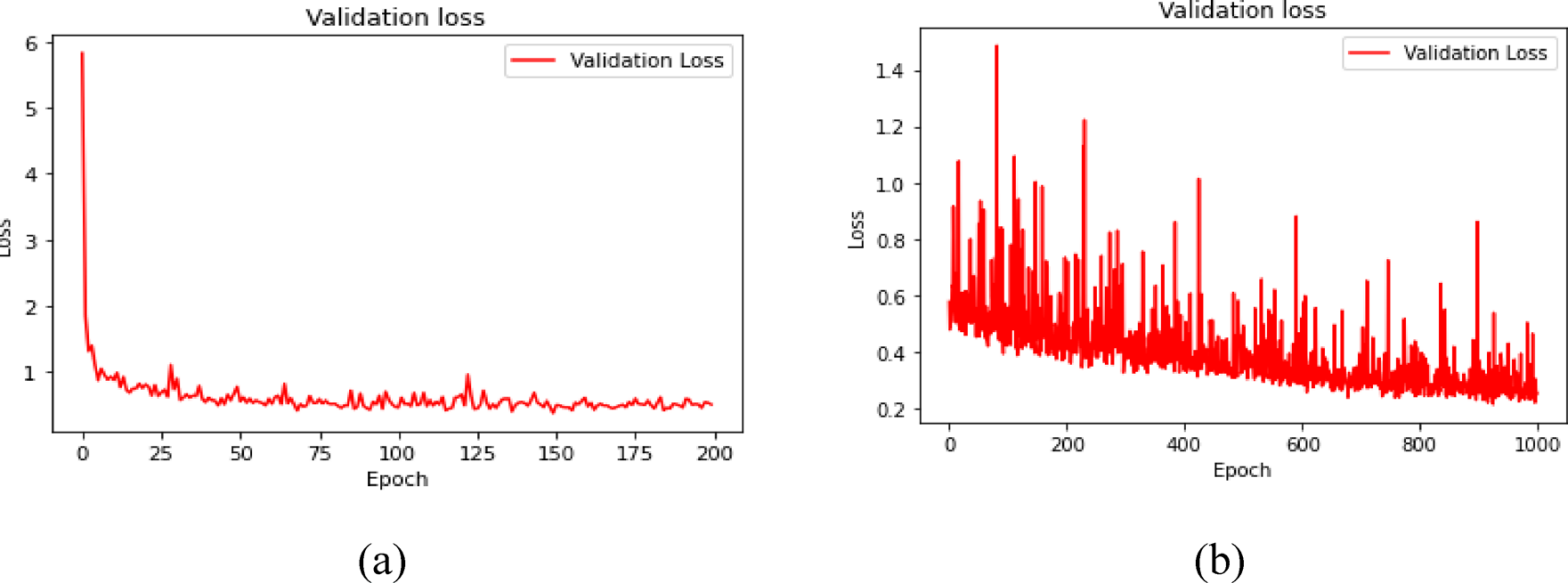
Epoch vs. Validation Loss for DNN (**a**) for S_11_ (**b**) for S_12_.

**Fig. 12:**
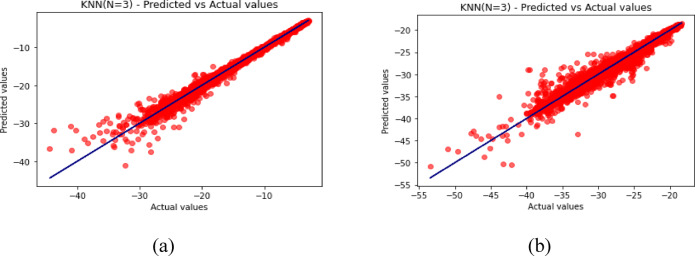
Predicted vs. Actual Values for KNN (*N* = 3) (**a**) for S_11_ (**b**) for S_12_.

**Fig. 13:**
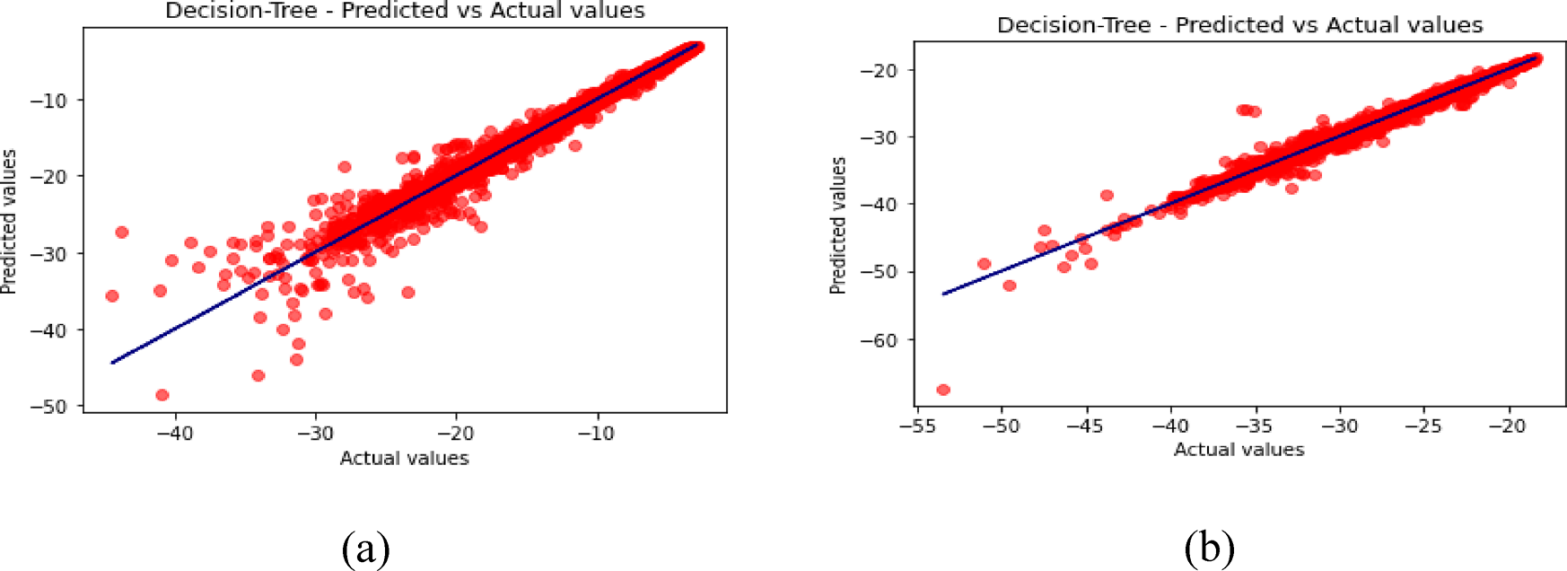
Predicted vs. Actual Values for Decision Tree (**a**) for S_11_ (**b**) for S_12_.

**Fig. 14:**
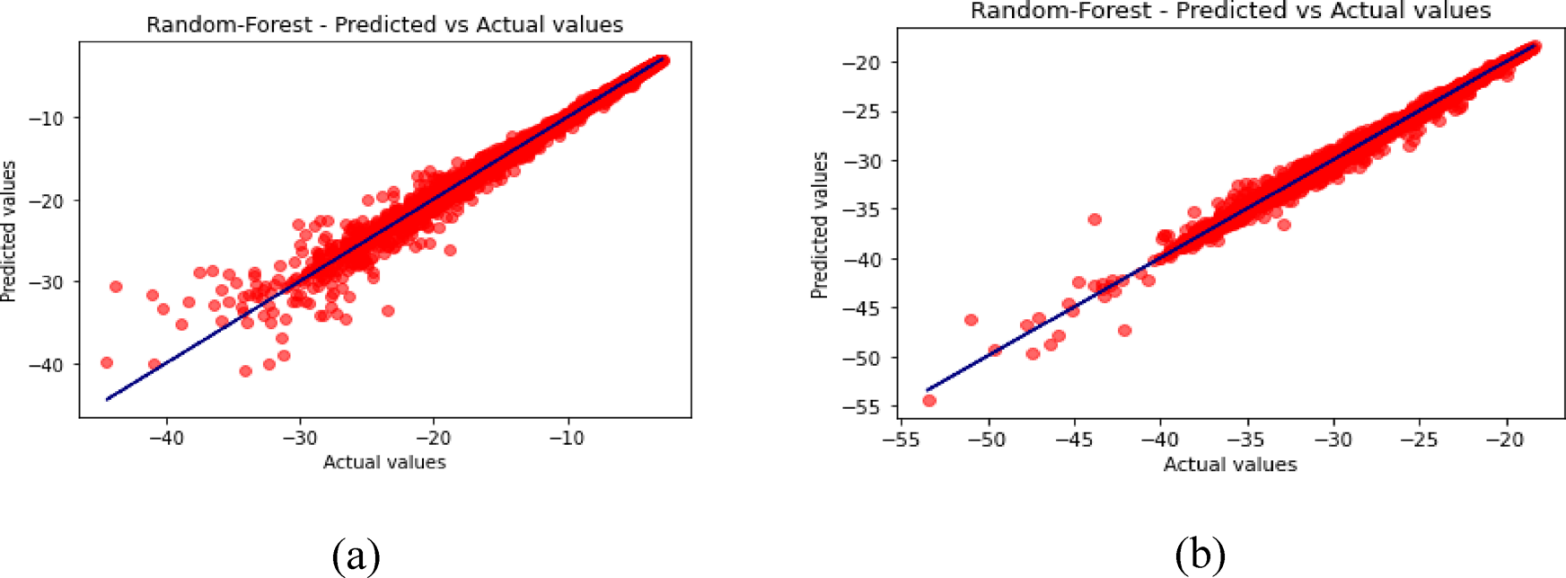
Predicted vs. Actual Values for Random Forest (**a**) for S_11_ (**b**) for S_12_.

**Fig. 15:**
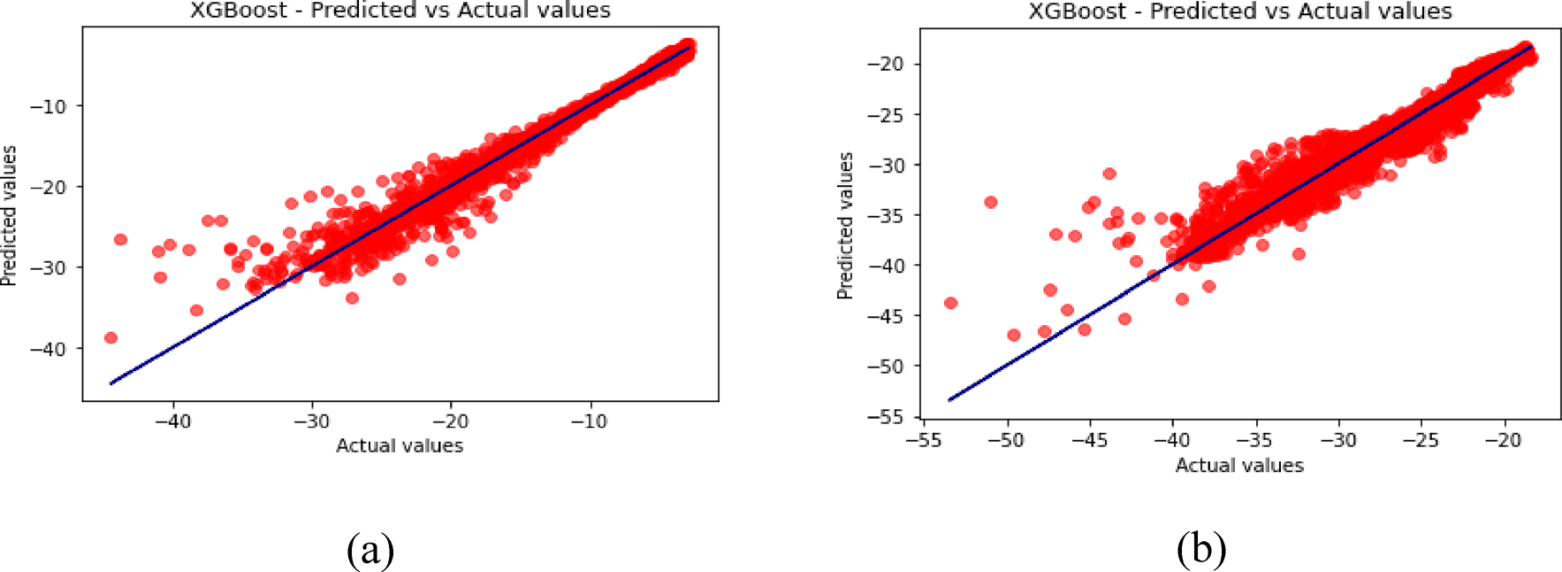
Predicted vs. Actual Values for XGBoost (**a**) for S_11_ (**b**) for S_12_.

**Fig. 16:**
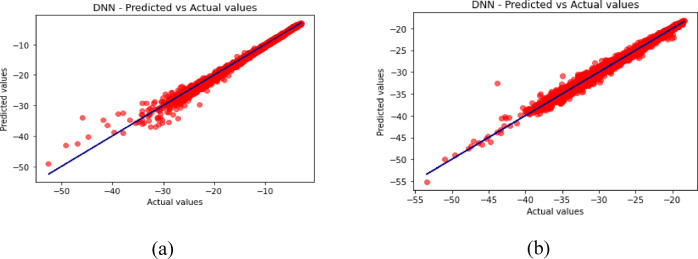
Predicted vs. Actual Values for DNN (**a**) for S_11_ (**b**) for S_12_.

**Fig. 17:**
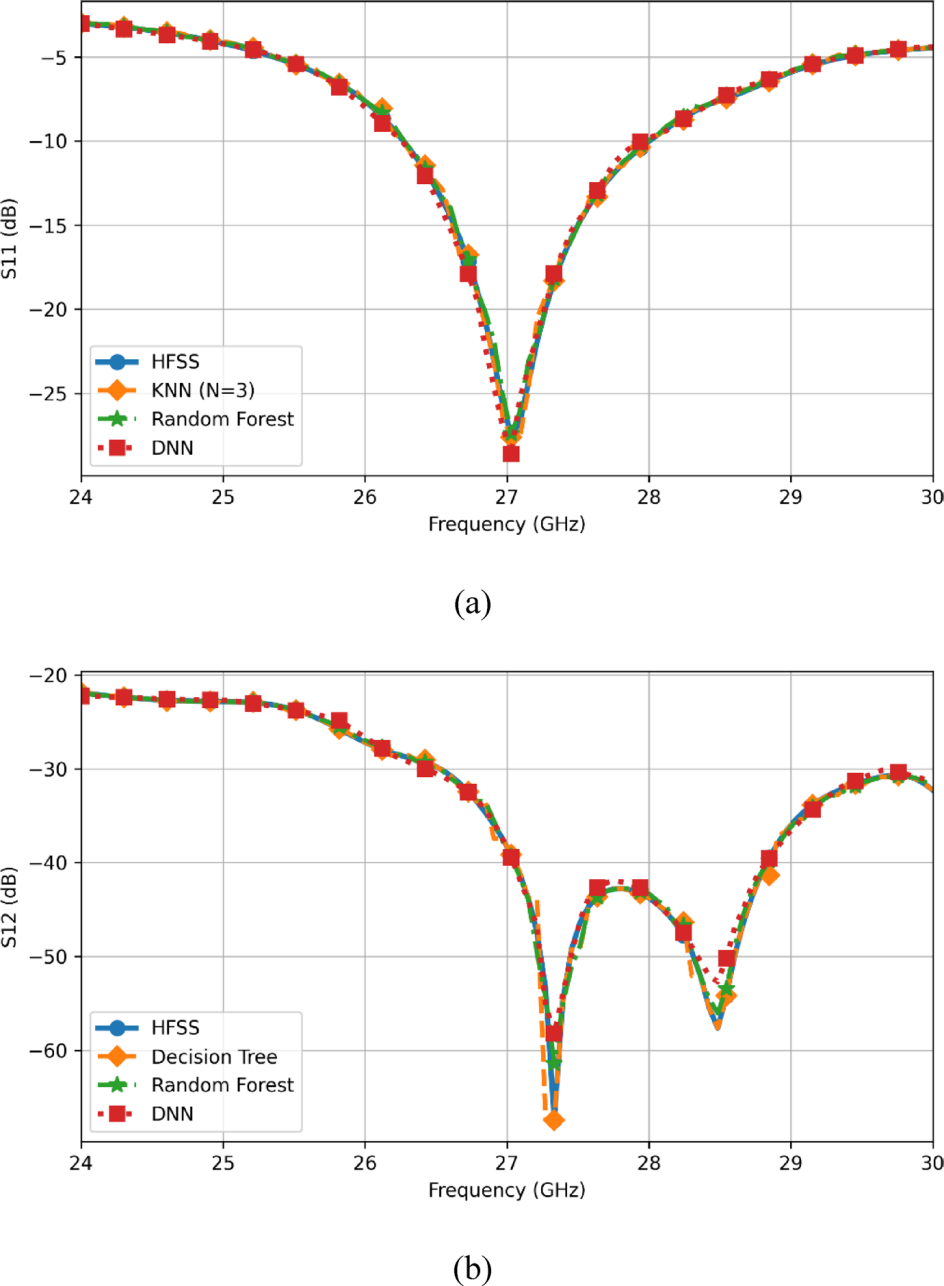
Comparison of different ML algorithms and HFSS simulated data (**a**) for S_11_ (**b**) for S_12_.

*How this work addresses the gap:* The present study proposes a dual-port cylindrical DRA-MIMO antenna around 27 GHz with a suspended CSRR metasurface to reduce mutual coupling (Figs. 1, 2, 3, 4, 5, 6, 7, 8 and 9). A dataset is generated in HFSS (Table 1) and used to train surrogate regression models to predict |S_11_| and |S_12_| across frequency and compare their performance (Tables 4, 5, Figs. 10, 11, 12, 13, 14, 15, 16 and 17). The antenna is fabricated and experimentally validated (Figs. 19, 20 and Fig. 21), and diversity parameters are reported (Table 6). Compared with earlier works, this paper keeps the antenna structure simple and uses a suspended CSRR metasurface to improve |S_12_| without disturbing |S_11_|. In addition, it presents an end-to-end workflow in which the antenna geometry is analyzed in HFSS, a dataset is generated, and surrogate models are trained to predict both |S_11_| and |S_12_| across frequency for faster parameter search, followed by hardware validation.

**Table 1:** General overview of the dataset.

	Height (h) [mm]	Length (l) [mm]	Freq. [GHz]	S_11_ [dB]	S_12_ [dB]
Count	19,100	19,100	19,100	19,100	19,100
Mean	8.096	4.036	27	− 9.213	− 25.910
Stdev	0.308	0.307	1.749	6.353	4.950
Min	7.609	3.7	24	− 52.56	− 67.461
Max	8.609	4.7	30	2.805	− 18.360

## Antenna design layout and unit cell

### Geometry of the proposed dual-port DRA-MIMO antenna

The configuration of the proposed dual-port dielectric resonator antenna (DRA) based MIMO structure is shown in Fig. 1. The antenna is designed on a Rogers RT/Duroid 5880 substrate of relative permittivity $\varepsilon_{r} = 2.2$ and thickness $0.254\;{\mathrm{mm}}$. The substrate size is $L_{s} = 11\;{\mathrm{mm}}$ and $W_{s} = 18.77\;{\mathrm{mm}}$, as illustrated in Fig. 1b.

Two identical cylindrical DR elements are used to form the dual-port MIMO antenna. The DR material is Rogers RT/Duroid 6010 with $\varepsilon_{r} = 10.2$. Each cylindrical DR has a height $H = 1.37\;{\mathrm{mm}}$ and diameter $D = 4.24\;{\mathrm{mm}}$ (Fig. 1b). The excitation is provided using identical microstrip feed strips for Port-1 and Port-2. The feed strip dimensions are $L_{1} = 6{\text{ mm}}$ and $L_{2} = 0.7{\text{ mm}}$. A horizontal slot is etched on the ground plane to support coupling to the DRA. The slot dimensions are $L_{{{\mathrm{slot}}}} = 0.3{\text{ mm}}$ and $W_{{{\mathrm{slot}}}} = 2.28{\text{ mm}}$. The fabricated prototype without and with the metasurface is shown in Fig. 2a and b, respectively.

### Metasurface concept and unit-cell design

To reduce mutual coupling between the two ports, a suspended metasurface is introduced above the MIMO antenna. The metasurface is implemented using a periodic arrangement of a complementary split-ring resonator (CSRR) type unit cell, as shown in Fig. 3. The unit cell is designed to operate around $27~\mathrm{GHz}$, which corresponds to the target operating band of the antenna.

The unit cell is realized on Rogers RT/Duroid 5880 ($\varepsilon_{r} = 2.2$, thickness $0.254\;{\mathrm{mm}}$). The rectangular CSRR geometry uses a strip thickness $t = 0.5\;{\mathrm{mm}}$ and a gap $d = 0.7\;{\mathrm{mm}}$, as marked in Fig. 3. The simulated reflection and transmission characteristics of the unit cell are presented in Fig. 4a. A clear resonance is observed close to $27\;{\mathrm{GHz}}$, and the transmission coefficient becomes very small around the same frequency, indicating a stopband-like behaviour that helps suppress coupling.

The effective material behaviour of the unit cell is summarized in Fig. 4b using retrieved effective parameters from the simulated response. Around $27\;{\mathrm{GHz}}$, the permittivity is negative while the permeability remains positive, which indicates single-negative behaviour. This property supports coupling suppression when the periodic surface is placed as a superstrate above the antenna elements.

### Metasurface array formation and placement

After finalizing the unit cell, a metasurface array is formed using a $4 \times 7$ periodic arrangement, as shown in Fig. 1c. The inter-element spacing is kept at $0.7\;{\mathrm{mm}}$. The overall metasurface dimensions are $S_{l} = 35{\text{ mm}}$, $S_{w} = 20{\text{ mm}}$, and thickness $S_{t} = 0.254\;{\mathrm{mm}}$.

The metasurface is placed above the dual-port antenna at a height $H_{1} = 8.49$ mm, as illustrated in the side view in Fig. 1d and the fabricated structure in Fig. 2b. This separation is a key design parameter because the coupling suppression depends on the strength of interaction between the metasurface and the antenna near-field region. Therefore, the value of $H_{1}$ is later optimized through mutual coupling analysis (Fig. 9) to obtain the best isolation improvement while keeping the impedance matching unchanged.

From a practical point of view, the suspended metasurface should remain mechanically stable so that the designed height $H_{1}$ and parallel alignment are maintained. In prototype fabrication, this can be achieved using low-permittivity dielectric spacers or a rigid dielectric support frame. Since the isolation improvement is sensitive to height error and tilt at mmWave frequencies, keeping the metasurface plane parallel to the antenna plane is important during measurement and for any practical integration.

## Proposed antenna analysis

### Single-element validation and mode identification

To confirm that radiation is mainly contributed by the dielectric resonator, the reflection coefficient of the single-port antenna is studied under two cases: with DRA and without DRA, as plotted in Fig. 5. When the DRA is removed, the resonance disappears, which confirms that the intended radiation is due to the dielectric resonator rather than the feed or slot structure.

The operating mode of the cylindrical DRA at 27 GHz is examined using the electric-field distribution shown in Fig. 6a and b. The field rotation and distribution indicate the excitation of the ${HEM}_{11\delta }$ mode inside the resonator, which is the desired dominant mode for this configuration^26^. The resonant frequency of the ${HEM}_{11\delta }$ mode is estimated using Eq. (1) based on standard cylindrical DRA relations^27^.

1 $$fr=\frac{6.324c}{2\pi D\sqrt{{\varepsilon }_{r}}}\left[1.22+3.56\left(\frac{D}{2H}\right)+0.002{\left(\frac{D}{2H}\right)}^{2}\right]$$

The calculated resonance is approximately $27.2~\mathrm{GHz}$, which is close to the simulated resonance observed in Fig. 5, validating the selected DRA dimensions.

The E-field plots in Fig. 6 confirm a dominant rotating field distribution inside the cylindrical DRA, which matches the expected behaviour of the HEM_11δ_ mode for this geometry. Also, the resonance obtained from Eq. (1) agrees well with the simulated resonance in Fig. 5, and no additional strong resonance appears within the operating band for the single element. The slot-coupled microstrip feed provides strong local coupling through the ground slot, which helps in exciting the fundamental mode when the slot location and DRA dimensions are designed for HEM_11δ_ operation.

### Dual-port MIMO behaviour without metasurface

Next, the single-port antenna is extended to a dual-port MIMO configuration by placing an identical element in parallel to form two ports. The simulated ${ \mid }S_{11} { \mid }$ and ${\mid }S_{12} {\mid }$ responses of this dual-port antenna without the metasurface are presented in Fig. 7. It is observed that the impedance matching remains almost unchanged after converting to two ports, indicating that the two elements are properly excited without strong mismatch. However, the mutual coupling is still noticeable, and ${\mid }S_{12} {\mid }$ is around $- 27.56{\text{ dB}}$ at $27{\text{ GHz}}$ (Fig. 7), which motivates the need for an effective decoupling approach.

### Effect of suspended metasurface on isolation improvement

After introducing the metasurface above the MIMO antenna, the simulated responses with and without metasurface are compared in Fig. 8. The main observation is that the metasurface does not significantly disturb impedance matching, as ${\mid }S_{11} {\mid }$ remains almost unchanged. This suggests that the main DRA resonance undergoes minimal mode loading and negligible frequency pulling at the optimized height $H_{1} = 8.49$ mm. At the same time, a clear improvement is achieved in mutual coupling: ${\mid }S_{12} {\mid }$ reduces from about − 27.56 dB to about − 45.22 dB at 27 GHz (Fig. 8). Therefore, the isolation improvement is approximately ${\Delta }{\mid }S_{12} {\mid } \approx \left( { - 27.56} \right) - \left( { - 45.22} \right) \approx 17.66$ dB.

In this work, the mutual coupling between ports is reported as ${\mid }S_{12} {\mid }$ in dB, while the isolation improvement is reported as the difference between the baseline case and the metasurface-loaded case at the same frequency. The coupling reduction is mainly due to the engineered CSRR response: the unit cell shows strong transmission attenuation near 27 GHz (Fig. 4a), which restricts field penetration through the superstrate region. When placed above the dual-port DRA, the metasurface supports induced currents that generate opposing fields to the coupling fields between the two radiators. As a result, the dominant near-field coupling path is weakened and ${\mid }S_{12} {\mid }$ improves, while ${\mid }S_{11} {\mid }$ remains stable. Similar metasurface-based decoupling behaviour has been discussed for MIMO systems where surface-current and near-field control reduces coupling between closely spaced radiators^28^.

### Optimization of metasurface height $H_{1}$

Since the decoupling strength depends strongly on the separation between the metasurface and the antenna, a parametric sweep is carried out by varying the antenna–metasurface distance. The resulting ${\mid }S_{12} {\mid }$ variation is shown in Fig. 9. From this analysis, the best mutual coupling suppression is achieved at $H_{1} = 8.49\;{\mathrm{mm}}$, which is therefore selected as the optimized placement height. When the metasurface is placed too close, strong interaction can alter the local fields and reduce the stability of the response. When placed too far, the metasurface influence on the coupling path becomes weaker, and the isolation improvement reduces. The selected height therefore provides a practical balance between decoupling strength and stable impedance behaviour.

## Machine learning optimization

Full-wave optimization at mmWave is computationally expensive because each change in geometry requires fine meshing and a dense frequency sweep. To reduce repeated EM simulations during tuning, this work uses surrogate regression models that learn the relationship between key design parameters and the antenna responses. Similar surrogate-assisted optimization has been reported as an effective way to speed up antenna design while maintaining accuracy when the surrogate is validated against full-wave results^16–18^.

### Dataset generation and features

After finalizing the antenna structure in HFSS (Sects. "Antenna design layout and unit cell" and "Proposed antenna analysis"), a dataset is generated by sweeping the most sensitive decoupling-related parameters:

- Metasurface height $h$ (antenna-to-metasurface separation $H_{1}$): 7.609 mm to 8.609 mm with a step of 0.05 mm.
- Unit-cell length *l:* 3.7 mm to 4.7 mm with a step of 0.05 mm.
- Frequency $f$: 24 GHz to 30 GHz with 100 points.

This selection is based on the physical role of the metasurface. The separation height $h$ controls how strongly the metasurface interacts with the antenna near-field region, and the sensitivity of ${\mid }S_{12} {\mid }$ with $h$ is already visible in Fig. 9. The unit-cell length $l$ controls the CSRR response near 27 GHz (Fig. 4) and therefore affects coupling suppression. Frequency is included so that the model predicts the full ${\mid }S_{11} {\mid }$ and ${\mid }S_{12} {\mid }$ curves from 24 to 30 GHz, rather than only a single operating point.

Using the above sweep, HFSS data are exported for ${\mid }S_{11} {\mid }$ and ${\mid }S_{12} {\mid }$ at each frequency point for every $\left( {h, l} \right)$ combination, resulting in a total dataset size of 19,100 samples. The overall dataset statistics are summarised in Table 1. A standard split is used, where 70% of samples are used for training and 30% for testing. In this work, ${\mid }S_{11} {\mid }$ and ${\mid }S_{12} {\mid }$ are treated as separate regression targets, and the input feature vector is $\left[ {h, l, f} \right]$.

Other parameters (feed width, slot size, metasurface spacing, array density, and DRA placement) also influence performance. In this manuscript, $h$ and $l$ are selected as the primary tuning variables for suspended metasurface decoupling while keeping the radiator and feed fixed for fabrication consistency and manageable dataset size. The same surrogate workflow can be extended to additional parameters when a larger multi-dimensional dataset is generated.

### Models and training settings

Five regression models are trained and compared: Decision Tree (DT), K-Nearest Neighbours (KNN), Random Forest (RF), Extreme Gradient Boosting (XGBoost), and a Deep Neural Network (DNN). The key training settings used for each model are summarized in Table 2 and the DNN architecture is summarized in Table 3.

**Table 2:** Key model settings used in this work.

Model	Main settings used
KNN	k = 3 neighbours, default distance metric
DT	default settings (baseline model)
RF	number of trees = 700
XGBoost	learning rate = 0.01, max depth = 8 (other parameters kept standard)
DNN	3 hidden layers, ReLU activation, Adam optimizer (learning rate = 0.001), MSE loss; epochs = 200 for $\mid {S}_{11}\mid$ and 1000 for $\mid {S}_{12}\mid$

**Table 3:** DNN model architecture summary.

Layer (type)	Output shape	Param #
Normalization_1(Normalization)	(None, 3)	7
Dense (Dense)	(None, 32)	128
Dense_1 (Dense)	(None, 64)	2112
Dense_2 (Dense)	(None, 64)	4160
Dense_3 (Dense)	(None, 1)	65

Training and testing split is kept as 70:30, and validation loss is monitored during DNN training. To explain the model behaviour, it is noted that ${\mid }S_{12} {\mid }$ is mainly controlled by the metasurface coupling mechanism, and therefore the geometry variables $h$ and $l$ are expected to have a strong impact on the coupling notch behaviour, as already indicated by the parametric trend in Fig. 9 and the unit-cell response in Fig. 4.

To evaluate the models, standard regression metrics are used: R^2^, MSE, RMSE, MAE, and MAPE. These metrics provide complementary views: R^2^ indicates overall explained variance, while MAE and RMSE quantify average prediction error in dB.

### Training behaviour and overfitting check

The training and validation loss curves of the DNN models are shown in Figs. 10 and  11 for ${\mid }S_{11} {\mid }$ and ${\mid }S_{12} {\mid }$, respectively. These plots show whether the model continues learning in a stable manner. The larger number of epochs for ${\mid }S_{12} {\mid }$ is used because coupling behaviour can be more nonlinear across frequency compared to impedance matching, and the regression surface may require additional iterations to converge. At the same time, long training can introduce overfitting if the validation loss stops improving.

### Test performance and model-to-model comparison

After training, each model is tested on the held out set of 5,730 samples. The quantitative comparison for ${\mid }S_{11} {\mid }$ is given in Table 4, and for ${\mid }S_{12} {\mid }$ in Table 5.

**Table 4:** Comparison of all models on performance metrics for S_11_.

Algorithm	R^2^ score	MSE	MAE	RMSE	MAPE
KNN (*N* = 3)	0.988	0.468	0.279	0.684	0.022
Decision Tree	0.978	0.877	0.387	0.936	0.03
Random Forest	0.986	0.547	0.306	0.740	0.024
XGBoost	0.977	0.908	0.412	0.953	0.038
DNN	0.991	0.363	0.314	0.602	0.030

**Table 5:** Comparison of all models on performance metrics for S_12_.

Algorithm	R^2^ score	MSE	MAE	RMSE	MAPE
KNN (*N* = 3)	0.966	0.824	0.500	0.908	0.017
Decision Tree	0.988	0.272	0.261	0.521	0.009
Random Forest	0.993	0.146	0.214	0.383	0.007
XGBoost	0.953	1.138	0.640	1.066	0.012
DNN	0.990	0.237	0.326	0.487	0.012

For ${\mid }S_{11} {\mid }$, the DNN achieves the best overall regression accuracy (R^2^ = 0.991), followed closely by KNN and Random Forest (Table 4). For ${\mid }S_{12} {\mid }$, Random Forest provides the best performance (R^2^ = 0.993), and DNN also performs strongly (R^2^ = 0.990) (Table 5). XGBoost shows weaker performance in the current setting, which suggests that further hyperparameter tuning (such as number of estimators, subsampling, and regularisation) may be required for this dataset.

The difference in model performance is mainly related to the response behaviour. The ${\mid }S_{11} {\mid }$ curve changes smoothly with frequency and geometry, so DNN can approximate the overall trend when sufficient samples are available. In contrast, ${\mid }S_{12} {\mid }$ is more sensitive to metasurface height near the coupling notch and shows stronger nonlinear variations, where an ensemble method like Random Forest can generalise better by averaging many decision trees. Overfitting is controlled by monitoring validation loss during training, and the final DNN model is taken at the minimum validation loss point.

The predicted-versus-actual plots in Figs. 12, 13, 14, 15 and 16 provide a visual check of regression quality. As expected, ${\mid }S_{12} {\mid }$ generally shows more scatter than ${\mid }S_{11} {\mid }$ because mutual coupling is influenced by a more complex interaction of fields between the two elements and the metasurface. This makes ${\mid }S_{12} {\mid }$ a harder regression target in comparison to reflection coefficient.

### Agreement with HFSS curves at a representative design point

Beyond scalar metrics, the practical usefulness of a surrogate model depends on how well it reproduces the S-parameter curves across frequency. Figure 17a compares ${\mid }S_{11} {\mid }$ versus frequency obtained from HFSS with the predictions from KNN, Random Forest, and DNN at $h = 8.49$ mm and $l = 4.3$ mm. Figure 17b provides the corresponding ${\mid }S_{12} {\mid }$ comparison for HFSS with Decision Tree, Random Forest, and DNN at the same parameter values. This figure confirms that the better-performing models (RF and DNN in particular) can closely follow the HFSS response, which supports their use as surrogates for fast parameter search.

### Surrogate-assisted optimization workflow

To convert prediction into optimization, the trained surrogate is used to quickly scan candidate parameter combinations before running a small number of verification simulations in HFSS. A practical optimization objective for this work is:

- Primary objective: minimize ${\mid }S_{12} {\mid }$ near 27 GHz (improve isolation).
- Constraints: maintain impedance matching in-band (${\mid }S_{11} {\mid } < - 10{\text{ dB}}$ within the target band) and keep the operating bandwidth aligned with the measured band.

Using this approach, a dense grid over $\left( {h, l} \right)$ is evaluated using the surrogate in seconds, and only the top candidate designs are re-simulated in HFSS for confirmation. This surrogate-assisted loop reduces the need for repeated full-wave tuning while keeping the final decision EM-verified, which is consistent with established practices in surrogate-based antenna optimization^16–18^.

## Measurement setup for proposed antenna

**Fig. 18:**
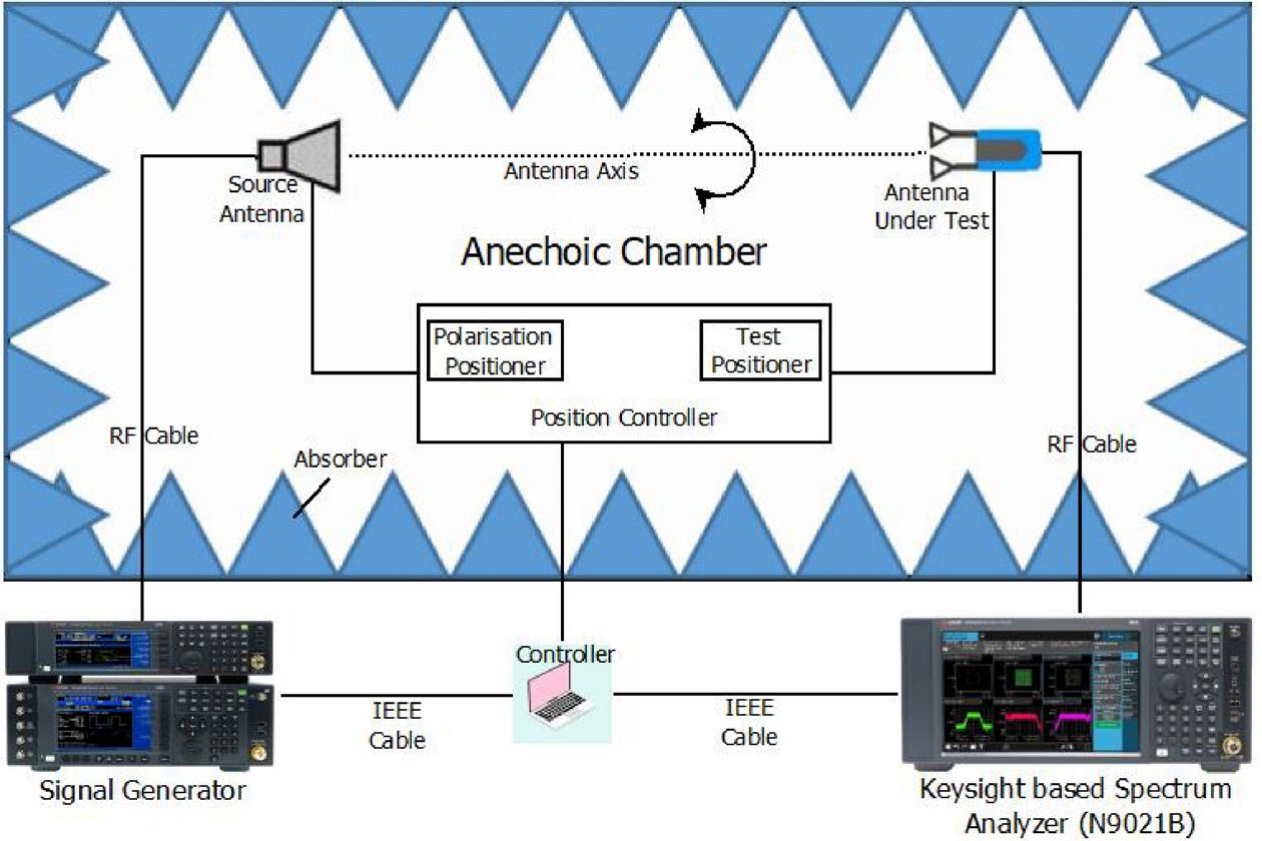
Measurement setup inside the anechoic chamber for mm wave MIMO antenna.

This section describes the measurement arrangement used to validate the proposed mmWave dual-port DRA-MIMO antenna. The complete setup inside the anechoic chamber is shown in Fig. 18. During measurements, one port of the MIMO antenna is terminated with a matched load to avoid reflections from the inactive port and to ensure consistent two-port behaviour.

### Radiation pattern measurement

A standard gain horn antenna is used as the transmitting antenna and is connected to the source (signal generator/VNA output, as available). The proposed antenna acts as the antenna under test (AUT) and is connected to the receiving instrument (spectrum analyzer/VNA receiver). The AUT is rotated through ${360}^{\circ }$ in the azimuth plane to record the angular response, as illustrated in Fig. 18. For the E-plane measurement, the horn is aligned for the required polarization, and for the H-plane measurement, the horn polarization is adjusted accordingly. The measured patterns reported later are compared with simulation at 27 GHz.

### Gain measurement using the two-antenna method

The gain is measured using the two-antenna method, following standard antenna measurement practice^29^. The procedure is:

1. The AUT is placed in the broadside direction facing the standard gain horn.
2. The separation distance $R$ is selected to satisfy far-field conditions at the operating band.
3. The transmission coefficient $\mid {S}_{21}\mid$ (or received power difference) is recorded across frequency.
4. Cable and connector losses are measured and accounted for using a direct-through connection.
5. The AUT gain is obtained using the Friis transmission relation^29^:
  2 $${P}_{r}={P}_{t}\hspace{0.17em}{G}_{t}\hspace{0.17em}{G}_{r}{\left(\frac{\lambda }{4\pi R}\right)}^{2},$$

where ${P}_{t}$ and ${P}_{r}$ are transmitted and received powers, ${G}_{t}$ is the known gain of the transmitting horn, ${G}_{r}$ is the gain of the AUT, $\lambda$ is the wavelength, and $R$ is the separation distance.

## Experimental outcome and diversity parameter

This section reports the measured S-parameters, gain, and radiation patterns of the fabricated prototype, followed by diversity evaluation using ECC and DG.

### Measured S-parameters and operating band

**Fig. 19:**
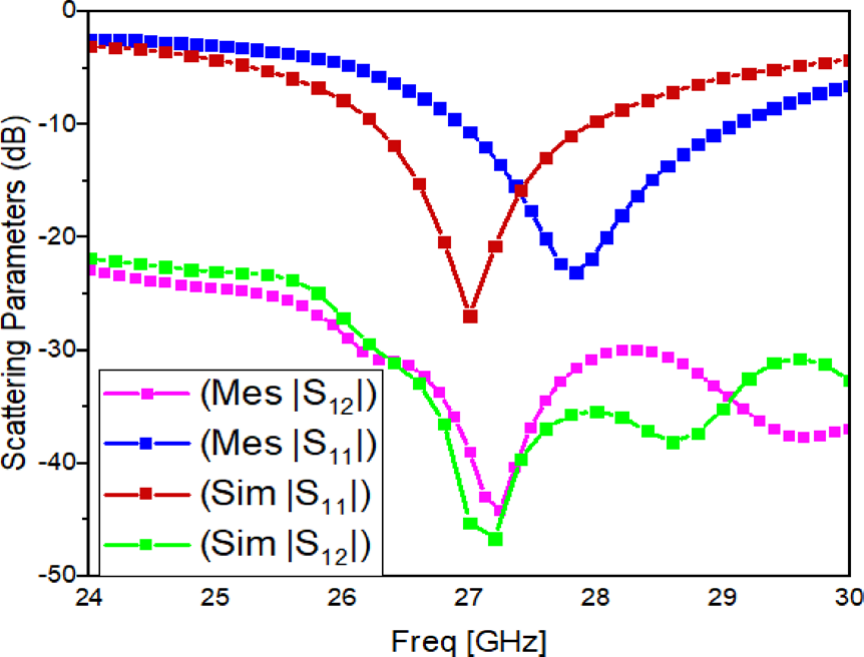
Simulated/Measured S-parameter of proposed dual-port antenna with metasurface.

The simulated and measured S-parameters of the fabricated dual-port antenna with metasurface are presented in Fig. 19. The measured $\mid {S}_{11}\mid$ confirms an operating band of 26.24–27.94 GHz, which agrees with the intended 27 GHz design target. A small shift between measured and simulated curves is observed. This mismatch is mainly due to small assembly tolerances at mmWave, including DRA bonding thickness, minor air gaps, slight metasurface height variation, and connector launch and calibration reference plane effects.

For isolation, simulation indicates strong coupling suppression when the metasurface is applied (as already shown in Fig. 8, where $\mid {S}_{12}\mid$ is reduced to about $-45~\mathrm{dB}$ near 27 GHz and the improvement is about $17.6~\mathrm{dB}$). In measurement (Fig. 19), the coupling level is lower than the simulated value. This reduction is expected because the metasurface-based decoupling effect depends strongly on precise spacing and alignment between the metasurface and the antenna elements. Even a small deviation in separation height or parallelism at 26–28 GHz can weaken the suppression mechanism.

Reasons for the simulated–measured isolation difference:

- *Metasurface height error and tilt:* The decoupling is optimized at ${H}_{1}=8.49~\mathrm{mm}$ (Fig. 9). Small height deviation or non-parallel mounting changes the near-field interaction.
- *DRA placement and adhesive layer:* The adhesive introduces an additional dielectric layer and possible air gaps, shifting the effective resonance and modifying coupling paths.
- *Connector and launch effects:* At mm-Wave, connector repeatability, soldering/launch geometry, and calibration plane placement affects measured $\mid {S}_{12}\mid$.
- *Fabrication tolerances:* Unit-cell etching tolerance and small dimensional variations change the metasurface resonance and reduce the stopband strength.

### Measured gain result

**Fig. 20:**
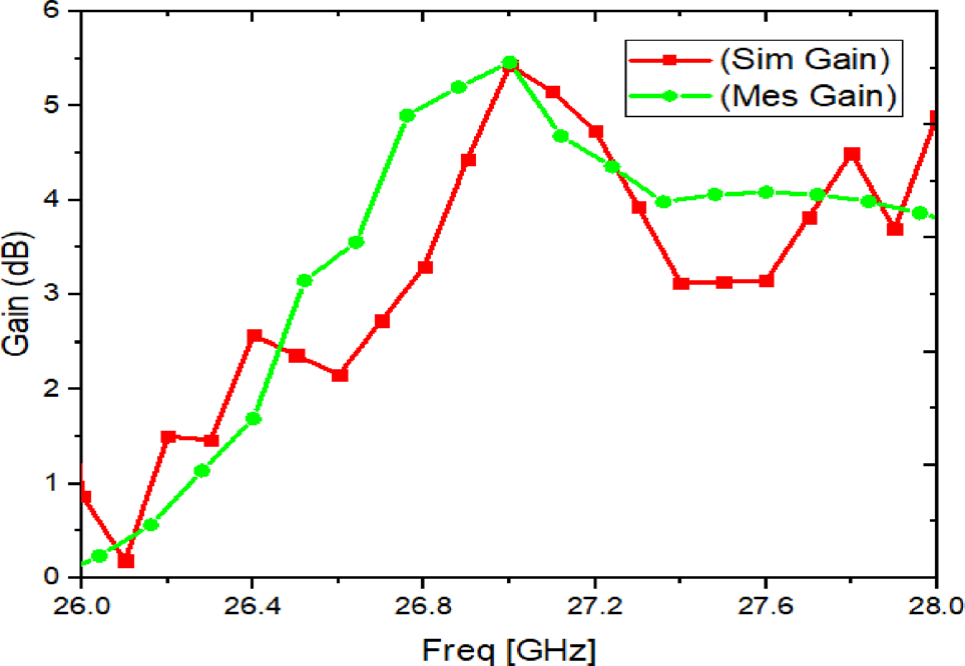
Simulated/Measured Gain of proposed dual-port antenna with metasurface.

The simulated and measured gain curves are shown in Fig. 20. The gain remains positive across the operating band and is around 5 dBi near the center frequency. The difference between simulated and measured gain can be linked to the same practical factors discussed above (connector loss, alignment, and assembly tolerances), as well as uncertainty in the two-antenna method calibration at mm-Wave^29^.

### Radiation pattern validation (XZ and YZ planes)

**Fig. 21:**
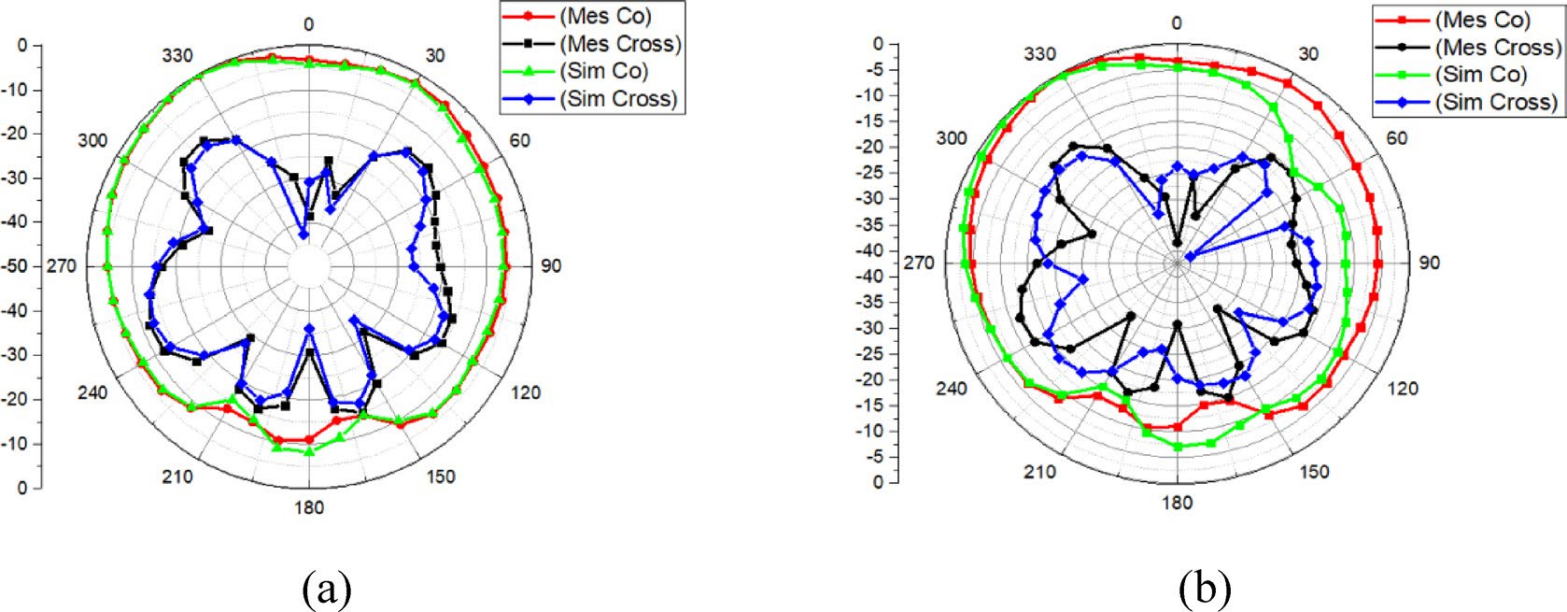
Measured and simulated radiation Pattern of proposed antenna at 27 GHz: (**a**) XZ-plane, (**b**) YZ-plane (Co-pol and Cross-pol).

The simulated and measured radiation patterns at 27 GHz are compared in two orthogonal planes. Figure 21a shows the co-polar and cross-polar patterns in the XZ plane, and Fig. 21b shows the corresponding results in the YZ plane. In both planes, the measured co-polar patterns remain broadside and follow the simulated trend with small deviations. The cross-polar level stays clearly lower than the co-polar level around the main beam region, which indicates stable polarization behaviour at the operating frequency.

The minor differences between simulation and measurement are expected at mm-Wave due to sensitivity to fixture alignment, connector repeatability, and small assembly tolerances, particularly in the metasurface spacing and DRA placement. Overall, the XZ- and YZ-plane results confirm consistent radiation characteristics in orthogonal planes at 27 GHz.

### Diversity performance (ECC and DG)

Envelope correlation coefficient (ECC) and diversity gain (DG) are important diversity indicators for MIMO antennas. These parameters are computed using far-field radiation data following standard practice^1^. The calculated ECC and DG values are reported in Table 6. Across the operating band, ECC remains within acceptable limits and DG stays close to $10~\mathrm{dB}$, which indicates good diversity behaviour for the two-port configuration.

**Table 6:** Calculated ECC and DG using far-field parameters.

Frequency (GHz)	ECC	DG (dB)
26.3	0.09	9.96
26.5	0.12	9.93
27	0.156	9.93
27.5	0.095	9.95
27.9	0.096	9.95

It may be noted that the suspended metasurface mainly reduces near-field coupling, but it can also introduce small changes in the far-field phase distribution because it behaves like a superstrate at mmWave frequencies. In this work, ECC is computed from far-field radiation data, and the obtained ECC values remain below 0.2 across the operating band (Table 6), which indicates low pattern correlation between the two ports. A detailed assessment separating pattern correlation and total efficiency would require additional measurements such as total efficiency or phase-centre analysis, which is kept as future work.

### Discussion summary

The measurements confirm three key outcomes:

1. The proposed antenna achieves the intended 27 GHz band with stable impedance matching (Fig. 19).
2. The metasurface-based structure improves isolation in principle (as demonstrated clearly in simulation, Figs. 8, 9) and provides measurable coupling reduction, although the measured isolation is lower than simulation due to mmWave tolerance sensitivity (Fig. 19).
3. The antenna maintains useful gain and a broadside radiation pattern with acceptable cross-polarization behaviour (Figs. 20, 21), and the diversity metrics meet standard expectations (Table 6)^1^.

### Comparison with recent state of the art

Table 7 compares the proposed antenna with representative recent and commonly cited mmWave MIMO works from the literature. The comparison focuses on operating band, number of ports, decoupling idea, coupling level ($\mid {S}_{12}\mid$), gain, and whether any ML or surrogate method is used.

**Table 7:** Comparison of the proposed work with related state of the art MIMO antennas.

References	Antenna/ports	Operating band (GHz)	Main decoupling idea	Reported isolation (dB)	Peak realized gain (dBi)	ML/surrogate used
^4^	2-port mmWave DRA MIMO	25.2–27.1 (H-plane), 25.4–27.0 (E-plane)	Metallic vias inserted inside DRA	> 30; 34.2 at 26 GHz (H-plane), ~ 50 max (E-plane)	4.7 (H-plane), 6.6 (E-plane)	No
^5^	2-port RDRA MIMO	27.25–28.59	Metal strip loading on resonator	> 24; maximum improvement 12 over 27.5–28.35	≈ 9.3	No
^6^	4-element DRA MIMO, 4 ports	26.71–28.91	Metamaterial printed on top of DRs	− 29.34 (with metamaterial), − 16.07 (without)	> 7	No
^14^	4-port mmWave MIMO antenna array	26 GHz band	Metasurface cover / superstrate	− 24 (abstract); 22 minimum (measured)	9.1 peak at 25.8 GHz	No
^20^	Dual-port wideband MIMO with metasurface absorber	32.5–42.5 (simulated), 32.9–43.0 (measured)	Deep-learning-optimised metasurface absorber	$\mid {S}_{12}\mid$ = − 26 dB (improved from − 15 dB without absorber)	5.5	Yes
^23^	4-port mmWave antenna array	25–30	DGS + interlinked CSRR + oblique slots + slot-loaded patches	Average isolation: $\mid {S}_{12}\mid$ = − 36, $\mid {S}_{13}\mid$ = − 40, $\mid {S}_{14}\mid$ = − 42	14 peak at 27.25 GHz; 12.4 average	No
^24^	4-port wideband mmWave MIMO	24–50	Metasurface + FSS stacked structure	Better than − 33; up to − 65 for some port pairs	10.35	No
This work	Dual-port cylindrical DRA MIMO + suspended CSRR metasurface	26.24–27.94 (measured)	Suspended CSRR metasurface superstrate	Improves from − 27.56 to − 45.22 at 27 GHz (≈17.6 dB improvement, simulation)	~ 5	Yes

Overall, the proposed antenna achieves strong simulated isolation at 27 GHz using a simple cylindrical DRA and a suspended CSRR metasurface, while the ML surrogate models accelerate the parameter search for $\mid {S}_{11}\mid$ and $\mid {S}_{12}\mid$ without repeated full-wave simulations.

## Conclusion

This work presented a dual-port cylindrical dielectric resonator (DR) based MIMO antenna with a suspended CSRR metasurface for mutual coupling reduction around 27 GHz. The proposed antenna covers 26.24–27.94 GHz in measurement. In simulation, the metasurface improves the mutual coupling level from about $\mid {S}_{12}\mid \approx -27.56$ dB to $\mid {S}_{12}\mid \approx -45.22$ dB at 27 GHz, which corresponds to an isolation enhancement of about 17.6 dB. The measured results confirm isolation improvement with the metasurface and show a realised gain of around 5 dBi in the operating band. Radiation patterns at 27 GHz are reported in both orthogonal planes (XZ and YZ) and show broadside behaviour with lower cross-polar levels compared to co-polar components.

To reduce the time required for mm-Wave tuning, surrogate regression models were trained using an HFSS-generated dataset (19,100 samples) with inputs $(h,l,f)$ and targets $\mid {S}_{11}\mid$ and $\mid {S}_{12}\mid$. Among the evaluated models, DNN provided the best prediction for $\mid {S}_{11}\mid$ $({R}^{2}= 0.991)$, while Random Forest and DNN achieved the strongest performance for $\mid {S}_{12}\mid$(${R}^{2}=0.993~\mathrm{and}~0.990)$. The predicted S-parameter curves from the better-performing models closely follow the HFSS curves at the representative design point, which supports their use for fast parameter screening before final EM verification.

The difference between simulated and measured coupling is mainly attributed to mmWave sensitivity to assembly tolerances, including metasurface height/tilt, DRA placement and bonding layers, and connector/launch effects. Overall, the combined metasurface-assisted decoupling and surrogate modelling approach provides a practical direction for designing compact high-isolation mm-Wave DRA-MIMO antennas with reduced optimization effort.

## Data Availability

The datasets used and/or analyzed during the current study are available from the corresponding author on reasonable request.
